# Engineering banana endosphere microbiome to improve Fusarium wilt resistance in banana

**DOI:** 10.1186/s40168-019-0690-x

**Published:** 2019-05-15

**Authors:** Yupei Liu, Aiping Zhu, Hongming Tan, Lixiang Cao, Renduo Zhang

**Affiliations:** 1 School of Environmental Science and Engineering, Guangdong Provincial Key Laboratory of Environmental Pollution Control and Remediation Technology, Sun Yat-sen University, Guangzhou, China; 2 School of Life Sciences, Guangdong Provincial Key Laboratory for Climate Change and Natural Disaster Studies, Sun Yat-sen University, Guangzhou, China

**Keywords:** ACC, Banana, Endophyte, *Enterobacter*, Ethylene, Fusarium wilt

## Abstract

**Background:**

Plant microbiome highlights the importance of endosphere microbiome for growth and health of the host plant. Microbial community analysis represents an elegant way to identify keystone microbial species that have a more central position in the community. The aim of this study was to access the interactions between the keystone bacterial species and plants during banana *Fusarium* wilt process, by comparing the endophytic bacterial and fungal community in banana roots and shoot tips during growth and wilting processes. The keystone bacterial species were isolated and further engineered to improve banana wilt resistance.

**Results:**

Banana endosphere microbiome structure varied during plant growth and wilting processes. Bacterial and fungal diversity in the shoot tips and roots increased with the development of the banana plantlets. The bacterial groups belonging to the *Enterobacteriaceae* family with different relative abundances were detected in all the samples. The *Klebsiella* spp. might be the keystone bacteria during the growth of banana plantlets. The relative abundance of *Fusarium* associated with the wilt disease did not increase during the wilting process. The endophytic *Enterobacteriaceae* strains *Enterobacter* sp. E5, *Kosakonia* sp. S1, and *Klebsiella* sp. Kb were isolated on *Enterobacteriaceae* selective medium and further engineered by expressing 1-aminocyclopropane-1-carboxylate (ACC) deaminase on the bacterial cell walls (designated as E5P, S1P, and KbP, respectively). Pot experiments suggested that plants inoculated with strains E5, E5P, S1, and S1P increased resistance to the *Fusarium* wilt disease compared with the controls without inoculation, whereas the *Klebsiella* inoculation (Kb and KbP) did not increase the wilt resistance. Compared with the inoculation with the wild strains E5 and S1, the inoculation with engineered strains E5P and S1P significantly increased wilt resistance and promoted plant growth, respectively. The results illustrated that the keystone species in the banana microbiome may not be dominant in numbers and the functional role of keystone species should be involved in the wilt resistance.

**Conclusion:**

The ACC deaminase activity of engineered bacteria was essential to the *Fusarium* wilt resistance and growth promotion of banana plants. Engineering keystone bacteria in plant microbiome with ACC deaminase on the cell walls should be a promising method to improve plant growth and disease resistance.

**Electronic supplementary material:**

The online version of this article (10.1186/s40168-019-0690-x) contains supplementary material, which is available to authorized users.

## Background

Banana (*Musa* spp.) is an important fruit and cash crop in the sub-tropic and tropic regions [1]. However, banana production is constrained by many soil-borne pests [2], and *Fusarium* wilt is one of the most destructive diseases of banana [2, 3]. The pathogenic *Fusarium oxysporum* f. sp. *cubense* (FOC) infects banana roots, and the infection progresses into vascularized portions of the rhizome in susceptible banana cultivars [2, 3]. Tyloses, gums, and gels are produced in xylem lumena in response to *Fusarium* infection, and the affected xylem becomes reddish and eventually pugged, impeding water and nutrient transport [3]. The production of fusaric acids by FOC causes yellowing of leaf lamina [3]. FOC can survive in the soil for decades and disseminate in diverse ways (i.e., infecting soil, surface water, plants, and others). With limited options to manage the *Fusarium* wilt, resistant cultivars are the only consistently effective tool for controlling the disease in pathogen infested soils. Nevertheless, resistant cultivars are commercially unacceptable, scarce, and nonproductive [3].

The tissue-culture technique has been widely employed for germplasm conservation and rapid clonal propagation of banana. By producing tissue-culture plants axenically in greenhouse and field, beneficial and nonpathogenic endophytes are excluded together with pests [4]. Manipulation of the plant microbiome can increase agricultural production, reduce the incidence of plant diseases, and reduce chemical inputs, resulting in more sustainable and effective agricultural practices [5–7]. Banana endophytes are beneficial to the host plant, including growth promotion, and biological control against plant viruses, nematode, and plant weevil [8–11]. Managing the banana endosphere microbial consortia isolated from the plant’s own microbiome may improve plant resistance to pathogens [12]. The traits make endophytes be potential natural resources for biological control of banana pests. Nonetheless, banana plants inoculated with endophytes are still infested by FOC in the field [10]. Genetically modified endophytes confer new characteristics of disease resistance to plants without direct manipulation of the host plant genome [13].

Banana tissue cultures are initiated from sucker-enclosed shoots after extensive surface sterilization and under aseptic conditions. Therefore, the tissue-cultured plantlets of banana may contain the crucial microbial members provided by shoot tips. The vertical transmission progress can select for crucial microbiota in banana, and the microbial species become a core component of the host plant microbiome [14, 15].

In plants, ethylene serves as signals for recognition of microbes [16]. The ethylene level during compatible or susceptible disease interactions may affect the development of disease symptoms [17, 18]. Bacterial 1-aminocyclopropane-1-carboxylate (ACC) deaminase can cleave ACC from plants, reduce the ethylene level in plant host, and thus promote plant growth [19]. Therefore, it is possible to improve the resistance to banana *Fusarium* wilt and plant growth, using engineered crucial microbiomes by expressing ACC deaminase on cell walls.

Deciphering the banana core microbiome and their correlations with host plant and pathogens is critical to utilize the microbiome to enhance plant growth and health. The objective of this study was to investigate the interactions between plants, fungi, and bacteria during wilting process using tissue-cultured plantlets (i.e., roots and pseudostems) and healthy and wilting mature banana plants. Based on comparative analyses of the banana endosphere microbiomes, subsequent functions of engineered crucial *Enterobacteriaceae* were evaluated for growth promotion and *Fusarium* wilt suppression of banana.

## Methods

### Sample collection and DNA extraction

Banana samples were collected from a banana plantation in the suburbs of Guangzhou, China (22° 55′ 238″ N, 113° 31′ 053″ E). These domesticated banana plants are dependent on propagation via cloning, either by using suckers and cuttings taken from the underground stem or through modern tissue culture. Most banana plantlets are produced by modern tissue culture. The transmission of microbiota to the progeny represents a way to ensure the presence of beneficial symbionts within the habitat. The banana symbiotic microbiota may transmit from shoot tips to explants and to mature plants. The samples in different growth stages (i.e., explants and healthy and wilting mature plants) were collected and analyzed simultaneously to avoid the sequencing deviation from different sequencing condition.

To determine crucial microbial members proliferated in plant tissues during the growth stages and wilting development, the pseudostems and roots of banana explants (*Musa* sp., AAA, Giant Cavendish cv. Baxi) on Murashige and Skoog (MS) basal medium supplemented with 5 m/L of benzyl aminopurine (BAP) (designated as Sstem and Sroot, respectively, *n* = 5) from Guangzhou Academy of Agricultural Sciences were collected for DNA extraction. Suckers of healthy and wilting mature plants (*Musa* sp., AAA, Giant Cavendish cv. Baxi) were collected from the banana plantation in the suburbs of Guangzhou, China (22° 55′ 238″ N, 113° 31′ 053″ E). The banana suckers were uprooted, the external leaf sheaths were removed, and the shoot tips (10–15 mm) with almost half shoot and corm tissues were excised aseptically. The shoot tips (0.1 g) were used for DNA extraction. The plant roots were collected in plastic bags, washed with tap water, and surface sterilized within 4 h [20]. The surface sterilized roots (0.1 g) were used for DNA extraction. Shoot tips and roots of healthy mature plant in the field (designated as Btip and Broot, respectively, *n* = 5), shoot tips and roots of wilting plants (designated as Wtip and Wroot, respectively, *n* = 5), and shoot tips and roots of plants adjacent to the wilting plants but without wilting symptoms (designated as Htip and Hroot, respectively, *n* = 5) were employed for total DNA extraction using E.Z.N.A. HP Plant DNA Kit (Omega) according to the manufactures’ protocol [20].

### Amplicon generation and Illumina MiSeq sequencing

The fungal primers ITS3 (5′-GATGAAGAACGYAGYRAA-3′) and ITS4 (5′-TCCTCCGCTTATTGATATGC-3′) targeting ITS2 *rRNA* genes were adopted to analyze fungal community [21]. The bacterial primers S17 (5′-CCTACGGGNGGCWGCAG-3′) and A21 (5′-GACTACHVGGGTATCTAATCC-3′) towards the bacterial 16S *rRNA* genes V3–V4 hyper variable region were selected for bacterial community analysis [22]. The sequencing libraries were generated following the previous methods [20]. The library quality was assessed with the Nanodrop2000c (Thermo Scientific, USA) according to the manufacturer’s instructions. The libraries were further sequenced by Genewiz Co. Ltd. (Suzhou, China) on Illumina MiSeq platform.

Raw sequences were merged by overlapping paired-end reads using FLASH software (v 1.2.7) [23]. The clean tags were obtained with the previous methods [24]. The UNITE database version 6 for Quantitative Insights into Microbial Ecology (QIIME) was used as a reference file for operational taxonomic unit (OTU) picking and community diversity analyses [22]. The tags with chimera were removed using UCHIME Algorithm [25, 26]. The effective sequences were grouped into OTUs using the UPARSE-OTU and UPARSE-OTUref algorithms of UPARSE software package at 97% sequence similarity (Uparse v7.0.1001) [27]. Finally, the Ribosomal Database Project (RDP) multiclassifier was used to define indicator species and assign representative sequences to the phylum and genus levels [28]. All the sequencing data and raw reads generated during this study were deposited in GenBank under the accession numbers of SRP055897 (16S) and SRP061527 (ITS).

### Isolation of core symbiotic bacteria and construction of surface displaying plasmids

The surface sterilized shoot tips were cut into small pieces (about 5 × 5 mm) and placed onto selective Eosin-Methylene Blue *(*EMB) medium with 2.0% agar. The shoot tip samples were incubated at 28 °C for 7 days for bacterial colony formation. The colonies were purified, identified, and transformed with surface displaying plasmids. The 16S *rRNA* gene sequences of isolated strains have been deposited in the NCBI GenBank database under the accession numbers of KY800390, KY8003901, and KY8003902. The surface displaying plasmids, consisting of promoter sequences from *Enterobacter cloacae*, ice nucleation protein-N gene *inaK*-N, ACC deaminase gene (*acdS* JQ646055), and pUC57, were constructed with the previous methods [29, 30] (Additional file 1: Figure S1). The plasmid was further transformed into isolated *Enterobacteriaceae* strains using BTX ECM 399 electroporators under the following conditions: 2.0 kV, 25 μF, 200 Ω, and 2.5 ms. Cells harboring the recombinant plasmid were grown in Luria-Bertani (LB) medium with 500 μg mL^−1^ ampicillin for 2 days. The cells were collected for determination of ACC deaminase activity by measuring the amount of α-ketobutyrate released [31]. Toluene was used to increase cell permeability by dissolving the lipid structure of cell envelope, which increased membrane fluidity.

### Biocontrol experiments

The indole-3-acetic acid (IAA) produced by endophytic bacteria in LB medium or LB medium supplemented with tryptophan (200 μg mL^−1^) was determined using the colorimetric method with Salkowski reagents [32]. The antagonistic activity of endophytic bacteria to *Fusarium* wilt pathogen was evaluated from plate confronting tests on banana pseudostem extract medium [33]. Endophytic bacterial sensitivity to fusaric acid (100 μg mL^−1^) was rated to be tolerant, moderately tolerant, and intolerant according to the inhibition percentages of fusaric acid by 0–40%, 41–80%, and 81–100%, respectively [34]. Strains tolerant to the concentration were rated as insensitive strains. Each treatment of the experiments was performed in triplicate.

Soil samples were collected from the banana plantation of Guangzhou, air dried, and sieved (5 mm). The soil was light gravel soil with texture of sandy loam (68% sand, 16% silt, and 16% clay). Inoculums of banana *Fusarium* wilt pathogen *Fusarium oxysporum* f. sp. *cubense* race 4 (FOC4) were prepared from potato dextrose broth and the soil was infested with 1% of the inoculums [33].

Banana explants were inoculated with endophytic bacteria by soaking in bacterial suspension (10^6^ cfu mL^−1^) for 2 h. Explants soaked in the sterile LB medium were used as the controls. The inoculated and control plantlets were planted in pots with the soil. One plantlet was sown in each pot and five pots were set up for each treatment. The soil moisture was kept at > 95% of saturation. The banana plants were kept in a glasshouse at 25 °C and day/night cycle of 16:8 h. The biocontrol effect was evaluated after 90 days of planting by measuring plant height, leaf area, pseudostem girth, and fresh and dry weights [35]. Disease index was evaluated based on the leave wilt index scale and vascular discoloration index [33]. The IAA and ethylene concentrations of banana leaves (0.1 g) were measured using the plant IAA and ethylene ELISA Kits (Kmsbiotech, Shanghai, China) according to the manufacturer’s protocol, respectively.

### Statistical analysis

OTUs using the QIIME software package at 97% sequence identity and phylogenetic relationships among the different microbial taxa were displayed by KRONA [36, 37]. Indices of Shannon, Chao1, Simpson, coverage, and ACE were calculated using Mothur to indicate the richness and diversity of the endophytic community in different samples [38].

All statistical analysis of data, including disease resistance, plant growth, ethylene concentrations, IAA concentrations, and enzyme activities, were performed using the SPSS statistical package (SPSS Inc., Chicago, USA). For the biocontrol experiments, analysis of variance (ANOVA) was conducted to compare the inoculation results among the different symbiotic bacteria. All statistical tests were subjected to ANOVA and the significance threshold was set up at *P* < 0.05.

## Results

### The diversity and richness of bacterial and fungal species

From the bacterial 16S *rDNA* V3-V4 region and fungal ITS2 sequencing, 20,012 bacterial processed reads were obtained with an average length of 430 bp and 113,780 fungal processed sequences with an average length of 338 bp for the samples (Additional file 1: Table S1). These bacterial reads could be assigned to 61 to 2601 OTUs per sample at the 97% identity. The number of fungal OTUs at 97% similarity ranged from 210 to 1915 in each sample. The bacterial and fungal species richness indices (i.e., Chao, ACE, Simpson, and Shannon indices) indicated that the roots contained more diverse bacteria and fungi than the shoot tips (Table 1). Although the tissue-cultured explants did not contact with exterior bacteria, the roots of explants contained more diverse bacteria than the pseudostems. The bacterial species richness indices in the shoot tips and roots increased with the growth of the banana plant. With the development of wilt disease, more diverse bacteria were detected in the shoot tips, whereas the roots of plants without wilting symptoms contained more diverse bacterial species than the roots of wilting plants. The roots of tissue-cultured explants contained more diverse endophytic fungi than the pseudostems. Contrary to endophytic bacteria, fungal species richness in the shoot tips and roots increased with the plant growth and wilt development.

**Table 1:** The alpha diversity indices of bacterial and fungal OTUs from samples of pseudostems and roots of explants (designated as Sstem and Sroot, respectively), shoot tips and roots of healthy banana plants in fields without wilting symptoms (designated as Btip and Broot, respectively), shoot tips and roots of wilting banana plants (designated as Wtip and Wroot, respectively), and shoot tips and roots of banana plants adjacent to the wilting plants but without wilting symptoms (designated as Htip and Hroot, respectively)

Sample	Bacterial OTUs					Fungal OTUs				
	Chao1*	ACE	Shannon	Simpson	Coverage	Chao1	ACE	Shannon	Simpson	Coverage
Sstem	147 ± 3.1	326 ± 5.4	2.85 ± 0.07	0.18 ± 0.01	0.85 ± 0.03	388 ± 4.8	596 ± 11.0	0.38 ± 0.01	0.86 ± 0.01	1.00 ± 0.00
Btip	182 ± 3.5	662 ± 11.1	3.54 ± 0.16	0.05 ± 0.00	0.64 ± 0.02	633 ± 5.7	900 ± 9.3	0.73 ± 0.03	0.72 ± 0.03	1.00 ± 0.00
Htip	250 ± 3.3	527 ± 13.0	3.62 ± 0.18	0.04 ± 0.00	0.60 ± 0.03	549 ± 4.0	811 ± 13.1	0.52 ± 0.03	0.80 ± 0.01	1.00 ± 0.00
Wtip	432 ± 4.5	447 ± 16.3	3.75 ± 0.15	0.03 ± 0.00	0.34 ± 0.01	689 ± 7.1	1305 ± 25.6	0.58 ± 0.01	0.79 ± 0.03	1.00 ± 0.00
Sroot	373 ± 4.1	621 ± 11.5	2.68 ± 0.09	0.24 ± 0.01	0.92 ± 0.03	950 ± 10.6	1421 ± 22.4	0.67 ± 0.02	0.81 ± 0.02	1.00 ± 0.00
Broot	382 ± 5.1	531 ± 13.2	3.14 ± 0.20	0.12 ± 0.00	0.94 ± 0.03	2805 ± 13.9	3658 ± 55.9	2.65 ± 0.07	0.28 ± 0.01	0.99 ± 0.01
Hroot	7443 ± 17.5	12576 ± 53.1	4.62 ± 0.24	0.14 ± 0.00	0.87 ± 0.02	2198 ± 7.3	3394 ± 39.7	2.00 ± 0.03	0.37 ± 0.03	0.99 ± 0.01
Wroot	3723 ± 13.7	6671 ± 23.1	4.05 ± 0.13	0.19 ± 0.00	0.85 ± 0.01	4182 ± 21.5	6436 ± 63.1	2.99 ± 0.06	0.16 ± 0.00	0.98 ± 0.02

*Both Chao1 and ACE described an estimate of the total number of phylotypes in a source environment, and Chao1 is particularly appropriate for data sets in which most phylotypes are relatively rare in the community, ACE is appropriate for data sets in which some phylotypes occur more frequently. Both Shannon and Simpson indexes comprehensively reflect the richness and evenness of community, while Shannon index is more sensitive to the richness of the community, and Simpson index is more sensitive to the evenness of the community. Coverage is a non-parametric estimator of the proportion of phylotypes in a library of infinite size that would be represented in a smaller library. Values are reported as mean ± SD (standard deviation) (*n* = 5)

**Fig. 1:**
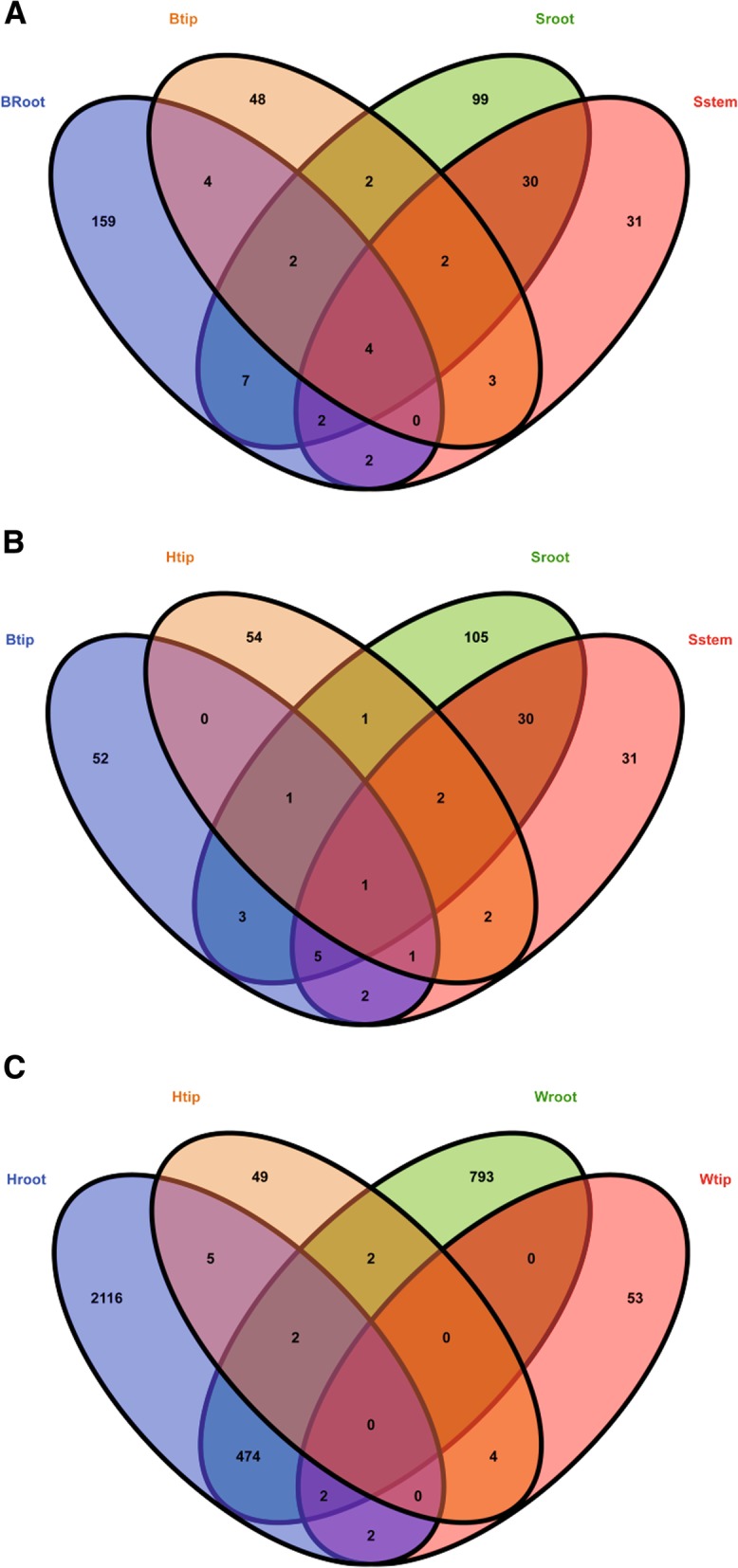
Venn diagrams of bacterial operational taxonomic units (OTUs) in samples of **a** pseudostems and roots of explants (designated as Sstem and Sroot, respectively) and shoot tips and roots of healthy banana plants in fields without wilting symptoms (Btip and Broot); **b** Btip, shoot tips of banana plants adjacent to the wilting plants but without wilting symptoms (Htip), Sroot, and Sstem; **c** shoot tips and roots of banana plants adjacent to the wilting plants but without wilting symptoms (Htip and Hroot), and shoot tips and roots of wilting banana plants (Wtip and Wroot). The Venn diagrams show the number of shared and unique OTUs in the samples. The different colored ovals represent different samples, and the intersections of the colored ovals indicate the coexisted bacterial OTUs in the samples

**Fig. 2:**
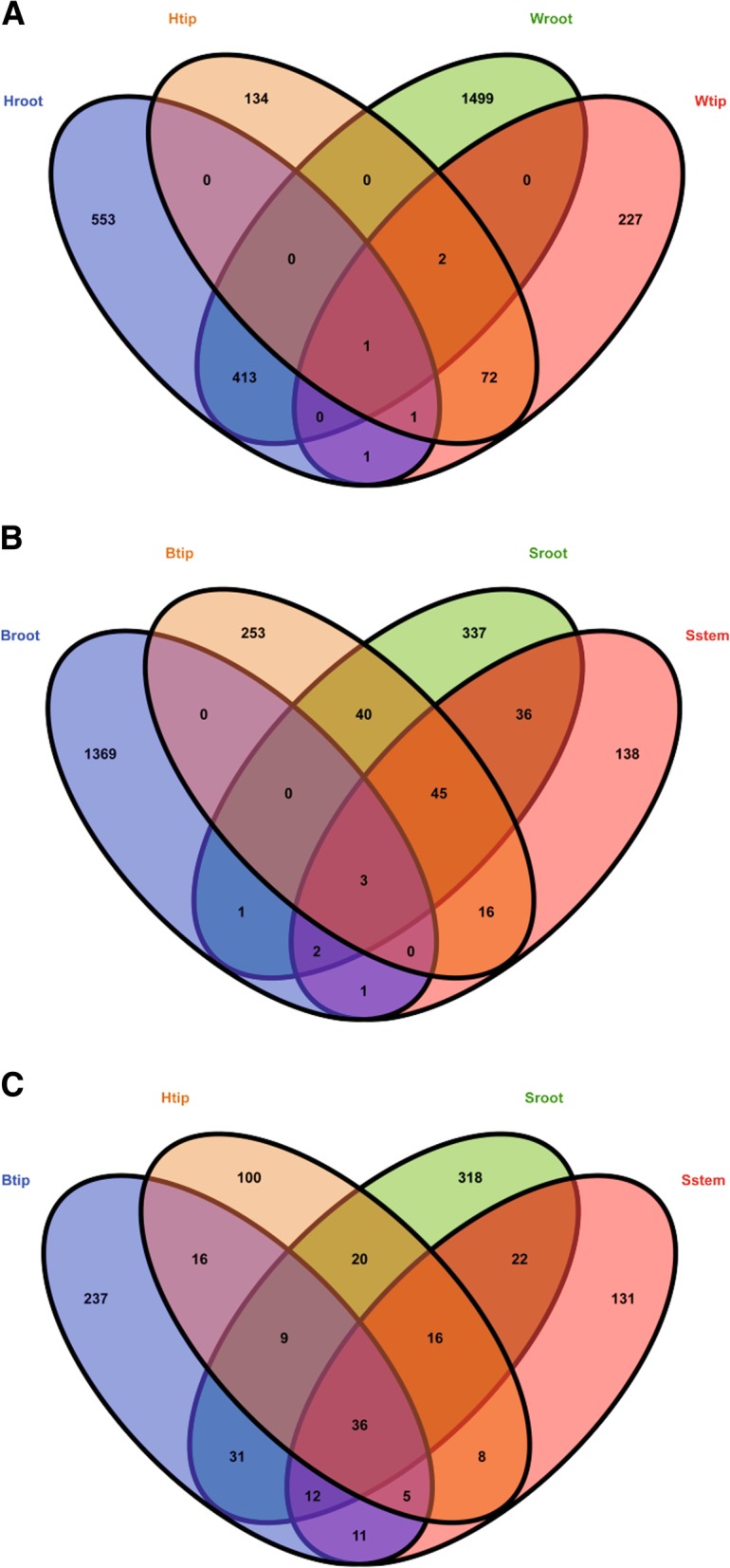
Venn diagrams of fungal operational taxonomic units (OTUs) in samples of **a** roots and shoot tips of banana plants adjacent to the wilting plants but without wilting symptoms (designated as Hroot and Htip, respectively) and shoot tips and roots of wilting banana plants (Wtip and Wroot); **b** pseudostems and roots of explants (Sstem and Sroot) and shoot tips and roots of healthy banana plants in fields without wilting symptoms (Btip and Broot); **c** Btip, Htip, Sroot, and Sstem. The Venn diagrams show the number of shared and unique OTUs in the samples. The different colored ovals represent different samples, and the intersections of the colored ovals indicate the coexisted bacterial OTUs in the samples

Tissue cultures were initially from sucker-enclosed shoots tips after following extensive surface sterilization. The tissue-cultured explants contained the crucial microbial members transmitted from shoot tips. The comparison of microbial flora in explants with that in mature plants revealed the microbial taxa proliferating in planta during the different plant growth stages. A total of 38 shared bacterial OTUs were detected in pseudostems and roots of the tissue-cultured banana explants (i.e., Sstem and Sroot, respectively) (Fig. 1a). The proportion of shared bacterial OTUs in pseudostems was higher than that in roots (51% vs. 26%). Compared with explants, the shared bacterial OTU number in mature plant roots and shoot tips (i.e., Broot and Btip) declined from 38 to 10. Nine shared bacterial OTUs were detected in the explant pseudostems and healthy plant shoot tips (i.e., Sstem and Btip) and 15 OTUs shared in the explant roots and healthy plant roots (i.e., Sroot and Broot). The shared bacterial OTUs still proliferated from the explant roots and pseudostems to the plant shoot tips and roots during the explant development. Four bacterial OTUs belonged to the core bacterial taxa of banana plants because they were detected in the different plant organs (i.e., pseudostems, shoot tips, and roots) from the explants to mature plants with different wilting processes (Fig. 1a). During the development of *Fusarium* wilt, the number of shared bacterial OTUs in the shoot tips of plants without wilting symptoms (Htip) declined to 7. The shoot tips shared 3, 5, and 6 bacterial OTUs with Btip, Sroot, and Sstem, respectively. Only one OTU was shared within the four types of samples (i.e., Htip, Btip, Sroot, and Sstem) (Fig. 1b). After the appearance of wilting symptoms, the shared bacterial OTU number in shoot tips and roots of wilting plants (i.e., Wtip and Wroot) was reduced to 2. Four OTUs still were detected in the plant shoot tips during the development of wilt symptoms (i.e., Wtip and Htip). The bacterial flora in roots varied slightly during the wilt development and 478 shared OTUs were detected in Wroot and Hroot (Fig. 1c). At the same time, 76 fungal OTUs were retained in the shoot tips (i.e., Wtip and Htip) and 414 OTUs in the roots (i.e., Wroot and Hroot) during plant wilting (Fig. 2a). Only one fungal OTU was shared in the shoot tips and roots during the wilting process, whereas the fungal OTUs were not affiliated with *Fusarium*. Consistent with bacteria, the banana explants contained 86 shared fungal OTUs in the roots and pseudostems (i.e., Sroot and Sstem) (Fig. 2b). The shared fungal OTU number in the healthy mature plant shoot tips and roots (i.e., Btip and Broot) declined to 3. During the sterile culture-tissue process, 48 fungal OTUs in the shoot tips (Btip) transmitted into the explant roots and pseudostems (i.e., Sroot and Sstem). Sixty-six fungal OTUs retained in banana shoot tips during the wilt pathogen infection (i.e., Btip and Htip), while the shared fungal OTU number in the shoot tips increased to 76 during the wilting symptom development (i.e., Wtip and Htip) (Fig. 2c). A total of 36 fungal OTUs in the explant pseudostems and roots (i.e., Sstem and Sroot) transmitted into the plant shoot tips during the processes of plant growth and infection by fungal pathogen (i.e., Btip and Htip) (Fig. 2). The banana plant also contained core mycobiome from explants to mature plants during fungal infection processes.

### Differences in fungal and bacterial community composition

**Fig. 3:**
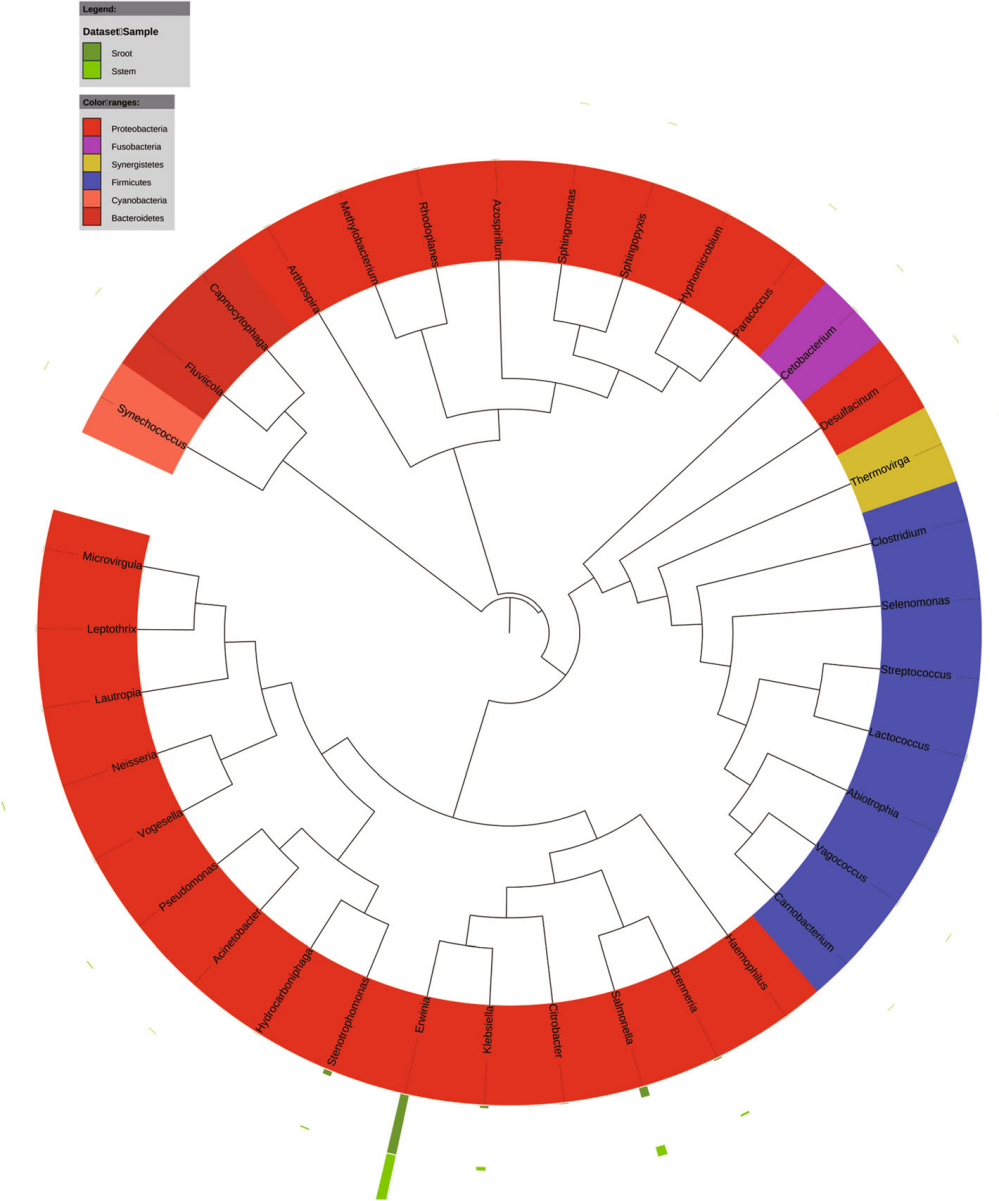
Phylogenetic analysis of bacterial operational taxonomic units (OTUs) in pseudostems and roots of explants (designated as Sroot and Sstem, respectively)

**Fig. 4:**
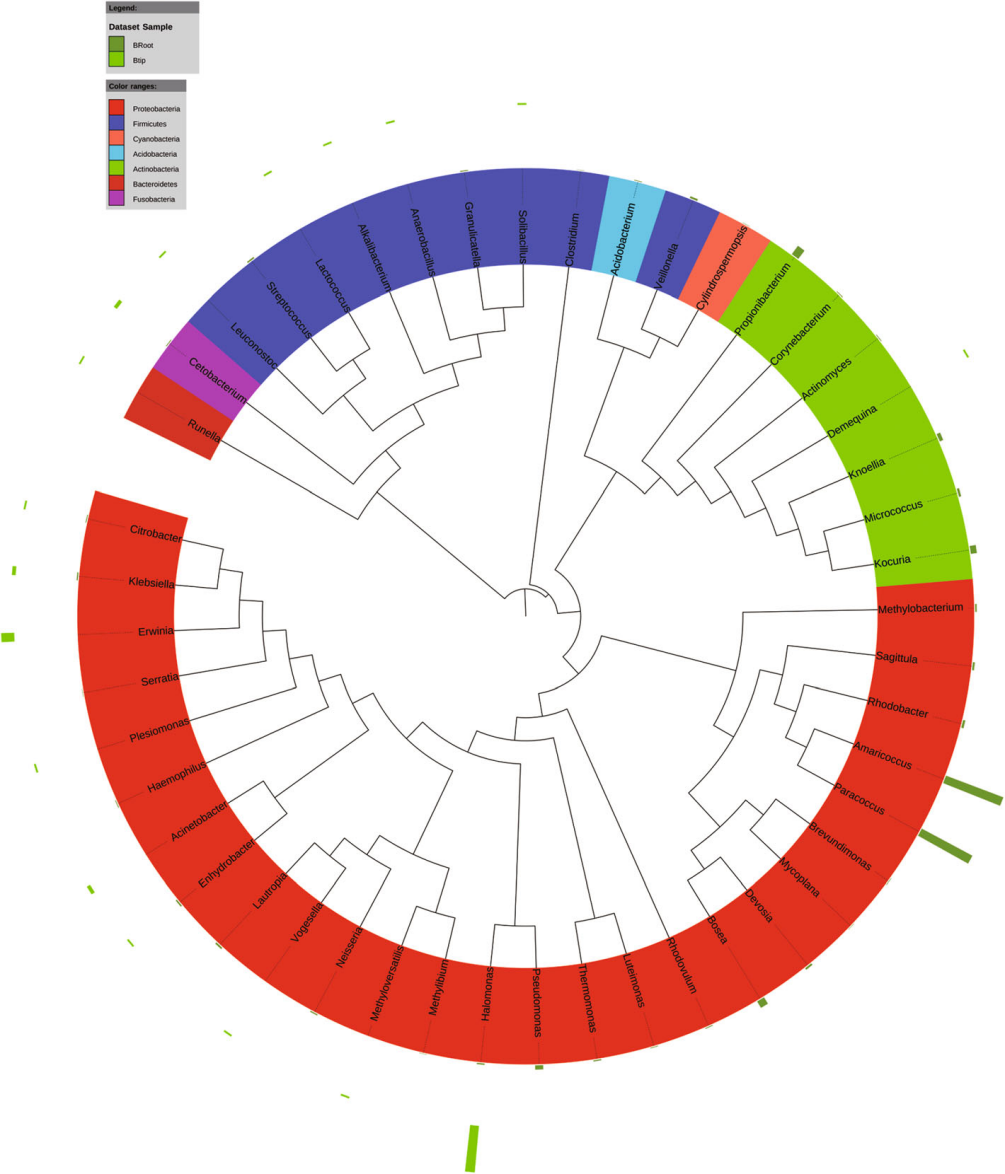
Phylogenetic analysis of bacterial operational taxonomic units (OTUs) in shoot tips and roots of healthy banana plants (designated as Broot and Btip, respectively)

**Fig. 5:**
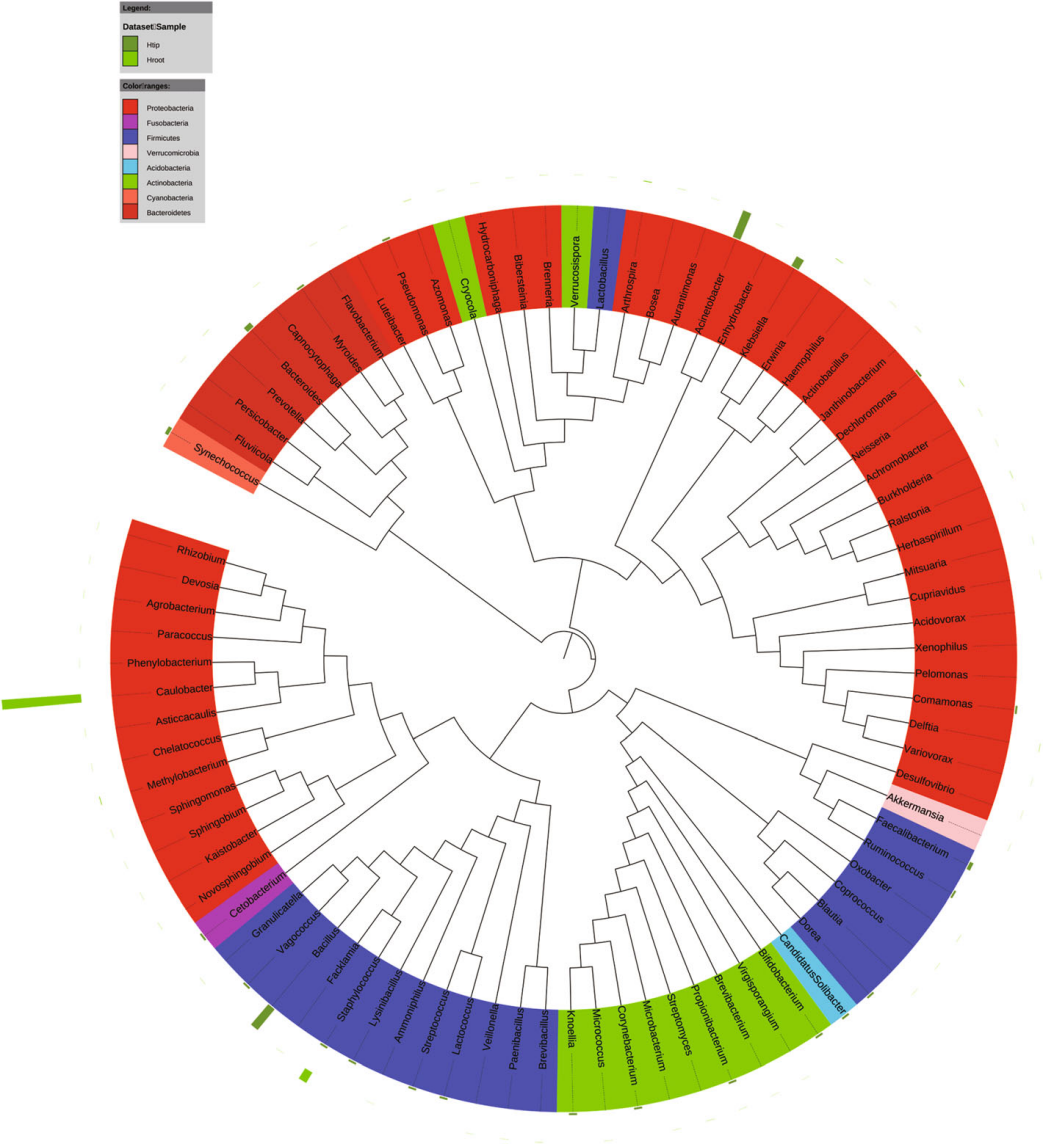
Phylogenetic analysis of bacterial operational taxonomic units (OTUs) in shoot tips and roots of banana plants adjacent to the wilting plants but without wilting symptoms (designated as Hroot and Htip, respectively)

**Fig. 6:**
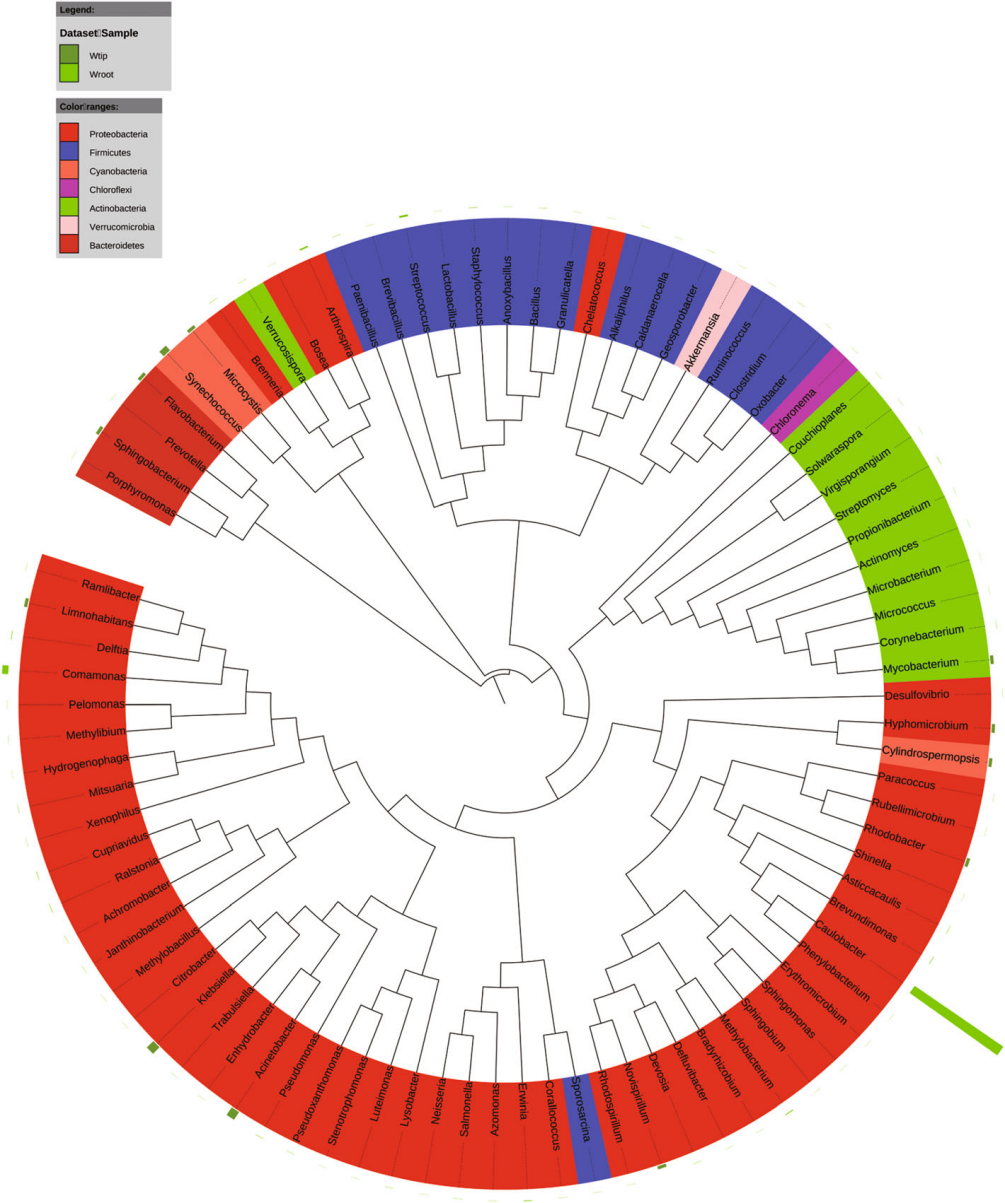
Phylogenetic analysis of bacterial operational taxonomic units (OTUs) in shoot tips and roots of wilting banana plants (designated as Wroot and Wtip, respectively)

At the genus level, *Caulobacter* (15%) and *Paracoccus* (2.9%) were the abundant bacterial genera in the banana plants. *Cladosporium* (12%) and *Eurotium* (4.2%) were the predominant fungal genera in the plants. *Erwinia*, *Salmonella*, *Klebsiella*, *Brenneria*, *Stenotrophomonas*, and *Lactococcus* were the most detected bacterial genera in the banana explants (Sroot and Sstem) (Fig. 3). In the mature plants, bacterial taxa *Halomonus*, *Erwinia*, *Klebsiella*, and *Citrobacter* dominated in the shoot tips (Btip), and *Amaricoccus*, *Paracoccus*, *Kocuria*, and *Micrococcus* were most frequently detected in the plant roots (Broot) (Fig. 4). Infested by fungal pathogen, *Acinetobacter*, *Bacillus*, and *Klebsiella* became the dominant bacterial taxa in the shoot tips (Htip), and *Caulobacter*, *Staphylococcus*, and *Comamonas* dominated in the roots (Hroot) (Fig. 5). With the development of wilting, *Acinetobacter* and *Klebsiella* were still the dominant bacterial genera in the shoot tips (Wtip), and *Caulobacter* and *Comamonas* were the dominant bacterial genera in the roots (Wroot) (Fig. 6). *Klebsiella* was considered as the crucial bacterial taxa in banana plant because it was detected throughout the plant growth and wilting processes.

**Fig. 7:**
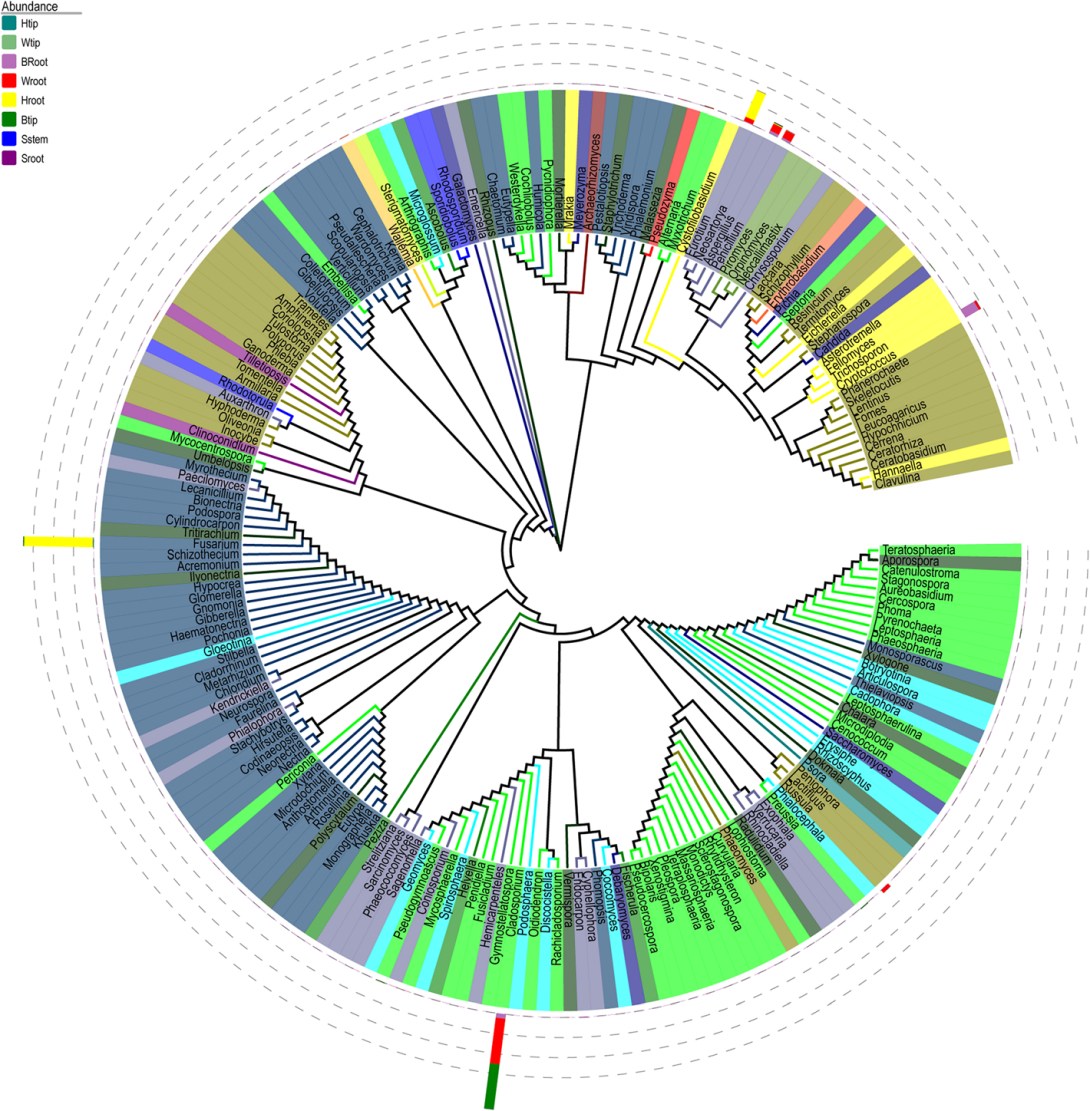
Phylogenetic analysis of fungal operational taxonomic units (OTUs) in samples of pseudostems and roots of explants (designated as Sstem and Sroot, respectively), shoot tips and roots of healthy banana plants in fields without wilting symptoms (Btip and Broot), shoot tips and roots of wilting banana plants (Wtip and Wroot), and shoot tips and roots of banana plants adjacent to the wilting plants but without wilting symptoms (Htip and Hroot)

**Fig. 8:**
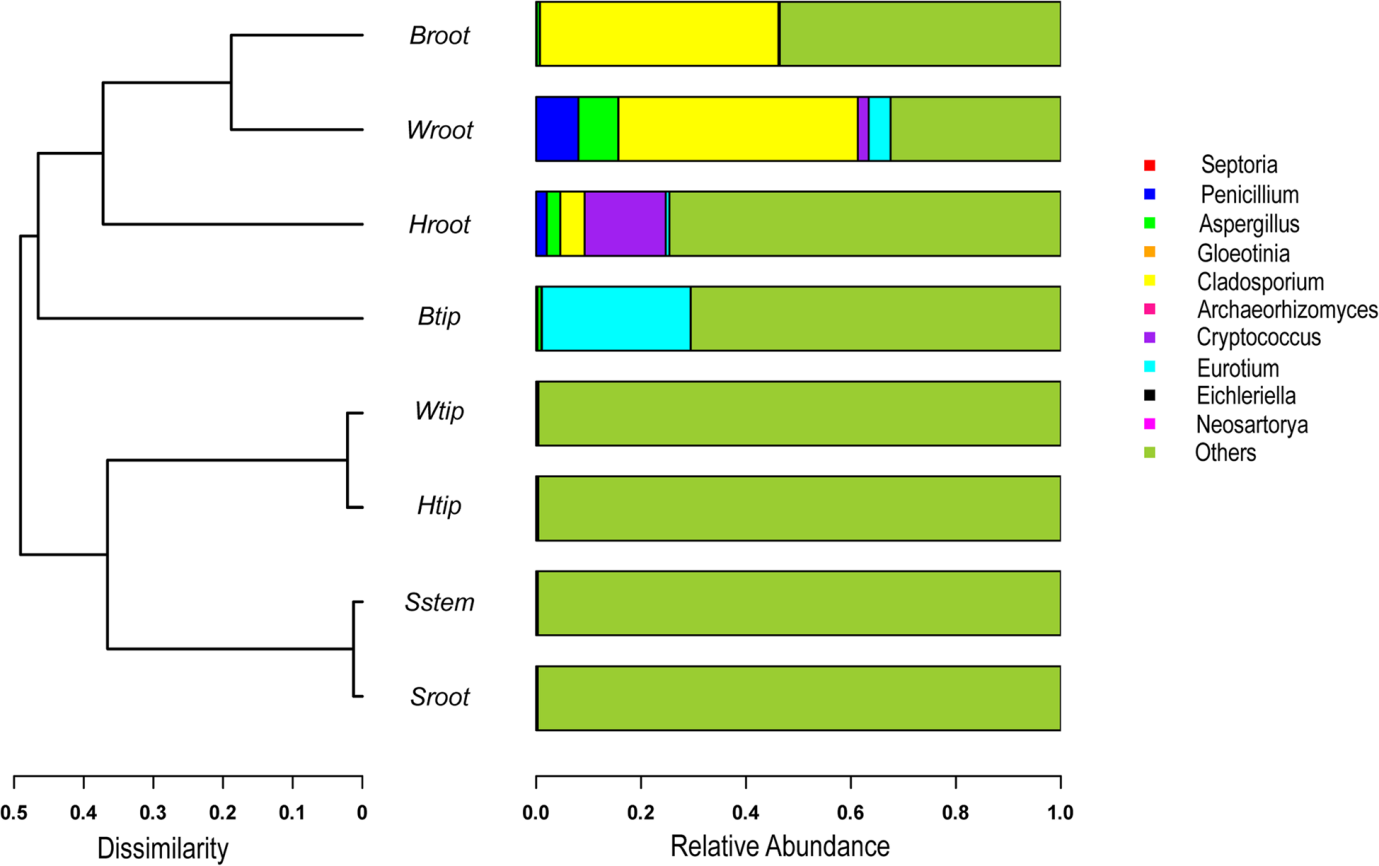
Phylogenetic dendrograms of endophytic fungal communities in samples of pseudostems and roots of explants (designated as Sstem and Sroot, respectively), shoot tips and roots of healthy banana plants in fields without wilting symptoms (Btip and Broot), shoot tips and roots of wilting banana plants (Wtip and Wroot), and shoot tips and roots of banana plants adjacent to the wilting plants but without wilting symptoms (Htip and Hroot). The same fungal genera are displayed in the same color in the figure

*Cladosporium* was the most frequently detected fungal genera in the healthy banana shoot tip (Btip). The fungal OTUs affiliated with *Fusarium* were not detected in the wilting plant roots, whereas *Cladosporium*, *Penicillium*, *Aspergillus*, *Eurotium*, and *Cryptococcus* were the dominant fungal genera in the roots (Wroot) (Fig. 7). *Cladosporium*, *Penicillium*, *Aspergillus*, and *Cryptococcus* might transmit from the explants because the fungal genera were more frequently detected in the explant roots (Sroot). During the infection process, *Fusarium* and *Eurotium* were detected in the roots of plants infested by the *Fusarium* pathogen (Hroot). With different relative abundance, *Eichleriella* was the crucial fungal taxa because it was detected in the pseudostems (shoot tips) and roots of explants and mature plants with different wilting stages (Fig. 8).

### Isolation of endophytic bacteria and transformed with surface display plasmids

The keystone endophytic bacterial genera *Klebsiella* belonged to the *Enterobacteriaceae*. Colonies grown from the shoot tip pieces were picked out, transmitted, and purified on selective EMB agar. The purified bacterial isolates were identified as *Kosakonia* sp. S1 (S1), *Enterobacter* sp. E5 (E5), and *Klebsiella* sp. Kb (Kb) by 16S *rRNA* gene sequencing analysis. The constructed surface display plasmids with ACC deaminase genes were transferred into E5, S1, and Kb cells with electroporation. The engineered strains of E5, S1, and Kb with plasmids were designated as E5P, S1P, and KbP, respectively. *Klebsiella* sp. Kb produced > 150 mg L^−1^ IAA, while E5 and S1 produced < 30 mg L^−1^ IAA in the media with and without tryptophan (Trp) addition. Plate confronting tests indicated that the strains of E5, E5P, S1, S1P, Kb, and KbP did not show any antagonism to the wilt pathogen FOC4 on the banana pseudostem extract medium. The growth of the six strains was not influenced by the addition of 100 μg mL^−1^ fusaric acid in vitro. These strains could tolerate the fusaric acid concentration of 400 μg mL^−1^ in the banana pseudostem extract medium. The bacterial strains could survive in banana plant tissues after the wilting symptoms appeared.

Without bacterial cells permeabilized with toluene, the ACC deaminase activities of the three wild bacterial isolates (i.e., E5, S1, and Kb) were similar, and the differences among the concentrations of α-ketobutyrate released from ACC were not significant (*P* > 0.05) (Table 2). The ACC deaminase activities of the isolates did not increase after the cells were permeabilized by toluene. The ACC deaminase gene transformed into *Klebsiella* sp. Kb did not express, as shown by the insignificant difference of ACC deaminase activities between Kb and KbP. The ACC deaminase activities of E5P and S1P permeabilized with toluene were 1.4 times higher than those of the corresponding strains without the toluene treatment. The most of ACC deaminase was expressed in cell envelopment interiors of E5P and S1P, and about 40% of the ACC deaminase was displayed on the cell walls of the strains.

**Table 2:** The deaminase activity of 1-aminocyclopropane-1-carboxylate (ACC) and the indole-3-acetic acid (IAA) concentrations in different strains of *Kosakonia* sp. S1 (S1), *Enterobacter* sp. E5 (E5), *Klebsiella* sp. Kb (Kb), and the engineered strains of E5, S1, and Kb (designated as E5P, S1P, and KbP, respectively) by expressing deaminase on the bacterial cells

	Concentration of α-ketobutyrate (mM)		Concentration of IAA (mg L^−1^) by Salkowski reagent	
	With toluene	Without toluene	Without Trp	With Trp
Control	0.00 ± 0.00b	0.01 ± 0.00b	0.33 ± 0.04c	0.41 ± .005c
S1	0.06 ± 0.01b	0.03 ± 0.01b	27.1 ± 1.33b	39.5 ± 3.46b
S1P	4.94 ± 0.35a	2.05 ± 0.13a	ND	ND
E5	0.06 ± 0.01b	0.05 ± 0.01b	22.6 ± 2.10b	45.1 ± 2.73b
E5P	4.82 ± 0.17a	1.96 ± 0.35a	ND	ND
Kb	0.04 ± 0.01b	0.03 ± 0.01b	155 ± 11.2a	161 ± 12.9a
KbP	0.11 ± 0.01b	0.08 ± 0.01b	ND	ND

The different letters within columns indicate that the values are significantly different at *P* < 0.05. Values are reported as mean ± SD (standard deviation) (*n* = 5)

*ND* not determined

### Effects of bacterial inoculation on growth and disease resistance of banana

After the plantlets were grown in the FOC4 infested soil for 90 days, the growth parameters (i.e., plant height, pseudostem girth, leaf area, and fresh and dry weights) of plants inoculated with the wild and engineered strains were compared. The growth parameters of plants inoculated with the six strains were improved compared with the controls without inoculation (Additional file 1: Figure S2A). The inoculation with Kb improved stem girth, leaf area, and fresh and dry weights of the plants and reduced the *Fusarium* wilt disease index by 10% (Table 3). The inoculation with E5 and S1 promoted the plant growth and reduced the *Fusarium* wilt disease index by about 25% and 40%, respectively. The inoculation with engineered strains E5P and S1P further improved the growth of banana plants and reduced the wilt disease index by 63% and 57%, respectively (Additional file 1: Figure S2B). The ACC deaminase of bacterial strains might be involved in the growth promoting and wilt resistance of banana plants (Table 3). The ACC deaminase on cell walls of endophytic strains could degrade ethylene synthesis precursor ACC and reduce ethylene levels in planta. At the same time, the plant IAA level increased. Inoculation with KbP without ACC deaminase activity did not reduce the ethylene level and not increase the plant IAA level compared with Kb (Table 3).

**Table 3:** Banana growth factors, ethylene concentrations, and indole-3-acetic acid (IAA) concentrations of banana of the control treatments (CK) and treatments with artificial inoculation of endophytes *Kosakonia* sp. S1 (S1), *Enterobacter* sp. E5 (E5), *Klebsiella* sp. Kb (Kb), and the engineered strains of E5, S1, and Kb (designated as E5P, S1P, and KbP, respectively) by expressing deaminase on the bacterial cells

Treatment	Plant height (cm)	Pseudostem girth (cm)	Leaf area (cm^2)^	Fresh weight (g)	Dry weight (g)	Disease index	Concentration of ethylene (nmol L^−1^)	Concentration of IAA (nmol L^−1^)
CK	16.03 ± 0.50e	4.07 ± 0.60d	91.53 ± 3.23d	41.73 ± 6.77d	13.50 ± 3.54c	62.67 ± 8.52a	89.51 ± 2.48a	60.09 ± 2.05e
S1	18.97 ± 0.60c	7.10 ± 0.30c	134.6 ± 10.57bc	59.93 ± 1.00c	22.17 ± 0.64b	33.67 ± 5.08e	71.90 ± 3.13 cd	68.01 ± 2.04 cd
S1P	21.17 ± 0.95b	9.30 ± 0.56a	208.0 ± 5.55a	102.6 ± 12.17a	37.40 ± 5.70a	26.67 ± 4.53f	63.70 ± 3.73ef	76.10 ± 2.75b
E5	17.77 ± 0.38 cd	6.60 ± 0.36c	122.9 ± 12.84c	58.07 ± 6.56c	22.03 ± 3.42b	47.00 ± 6.00d	69.51 ± 3.13de	70.19 ± 1.82c
E5P	23.63 ± 1.25a	8.43 ± 0.15b	207.3 ± 6.99a	111.2 ± 12.86a	41.53 ± 5.52a	23.33 ± 4.58f	57.94 ± 2.90f	80.57 ± 1.40a
Kb	16.67 ± 0.60de	7.03 ± 0.60c	138.8 ± 6.69b	74.40 ± 5.22b	27.50 ± 0.75b	56.00 ± 7.00b	82.92 ± 5.77b	65.99 ± 1.34d
KbP	16.83 ± 1.47de	7.43 ± 0.35c	144.8 ± 10.61b	74.77 ± 3.25b	27.70 ± 1.40b	51.67 ± 6.53c	77.94 ± 4.33bc	66.96 ± 1.82 cd

The different letters within columns indicate that the values are significantly different at *P* < 0.05. Values are reported as mean ± SD (standard deviation) (*n* = 5)

## Discussion

Despite decades of research, few effective methods are available to manage the banana *Fusarium* wilt disease. Resistance cultivar breeding is the best management method in affected areas. However, such bio-resources are often scarce, nonproductive, and commercially unacceptable [3]. It is difficult to create resistant cultivars using genetic transformation via the conventional breeding. Biocontrol using indigenous and disease-suppressive microorganisms can provide potential perspectives for sustainable plant protection [1]. Some antagonistic bacterial and fungal isolates from root endosphere and rhizosphere have been tested to control *Fusarium* wilt [1, 33]. In this study, the endophytic bacterial and fungal community variations were compared during banana plant growth, fungal infection, and wilting process. The fungal species richness (i.e., OTUs) in shoot tips increased with the banana plant growth and wilting development. The relative abundance of fungal OTUs affiliated with *Fusarium* did not increase during the wilting process. The wilting symptoms of banana plants were not correlated with the overgrowth of banana wilt pathogen in plant tissue but might be related to the ethylene level induced by pathogenic fungi [17, 18]. Therefore, the antagonistic activities of biocontrol agents towards *Fusarium* pathogen is not necessary to control banana *Fusarium* wilt and effective fungicides are not available to control the wilt disease nowadays [3]. The three *Enterobacteriaceae* isolates without antagonism to wilt pathogen promoted the growth and disease resistance of banana plants in our study. The antagonism to pathogenic fungi in vitro was not essential in selection for biocontrol agents. However, the tolerance to fusaric acid was essential because that fusaric acid played a critical role in accelerating the development of *Fusarium* wilt and inhibiting the growth of biocontrol strain by acting as a phytotoxin [34, 39].

Most studies on plant microbiome focused on rhizosphere bacteriome [6]. The endosphere bacteriome in banana shoot tips and plant mycobiome during plant growth and wilting development should be further studied. The seated shoot tips inside layers of leaf sheaths are usually used as starting material for micropropagated banana explants. The tissue-cultured explants may contain a genotype-specific core banana microbiome transmitted from shoot tips of last generation [40]. Since the explants grew under an aseptic condition, the roots differentiated from embryogenic suspension cultures of banana might not contact with exterior microbes. The bacterial and fungal florae in the explant pseudostem were similar to those in the explant roots. During plant growth, the banana bacterial and fungal florae might transmit between shoot tips and roots. Therefore, the core bacteriome and mycobiome could be detected in mature plant root interiors.

Plant core microorganisms establish consistently in plants and are not influenced by different spaces, time, and plant organs [16]. *Caulobacter* and *Paracoccus* were the most abundant bacterial genera in some banana samples. However, they were not considered as the crucial core bacterial endophytes because they were not detected in all the samples during the plant growth and wilt development processes. With lower relative abundance, *Enterobacteriaceae* endophytes of *Enterobacter* spp. and *Klebsiella* spp. were considered as the core bacterial taxa in banana because they were detected in roots and pseudostems (shoot tips) of banana explants and plants in the different wilting processes. Endophytic *Enterobacteriaceae* including *Klebsiella* genera have been detected from different tissues of banana with different methods [1, 40–45]. *Enterobacteriaceae* (*Enterobacter* spp. and *Klebsiella* spp.) may proliferate in plant tissues and transmit from generation to generation in banana growth and proliferation. *Klebsiella* and *Enterobacter* are more beneficial to banana plants than other microbial taxa [46]. Therefore, *Enterobacteriaceae* bacteria were considered as the keystone bacteriome in banana plants, whereas the keystone bacteria may not be dominant in numbers [47].

Although the core bacterial and fungal communities in banana plants have been elucidated, there is still a lack of knowledge about the relationships between endophytes and host plants [48]. In plants, IAA produced by endophytic bacteria serves as signals that intense plant-microbe interplay [15]. As shown in Tables 2 and 3, Kb produced fivefolds more IAA than E5 in the medium. However, the IAA contents of plantlets inoculated with Kb and KbP were lower than that with E5 (Table 2). The inoculation of IAA-producing Kb and KbP into plantlets did not increase IAA contents in planta. The engineered strain KbP without ACC deaminase activity did not reduce the ethylene level and not increase IAA contents in planta (Table 3). Therefore, the IAA increase in planta was correlated with bacterial ACC deaminase activities but not correlated with bacterial IAA production in the medium [49]. The increase in IAA contents in planta might be produced by host plants. The Salkowski reagents are specific for indoleacetamide, indolepyruvic acid, and IAA rather than for IAA alone [32]. Thus, the Salkowski reagents are not suitable for screening plant beneficial bacteria in vitro.

It remains difficult to correlate microbial OTUs with physiology nowadays [16]. In this study, the isolated *Klebsiella* and *Enterobacter* strains did not show any ACC deaminase activity in vitro. The *Klebsiella* and *Enterobacter* strains were further engineered by expressing ACC deaminase activity on the bacterial cells because the ACC produced by plants can contact with ACC deaminase on the endophytic bacterial cells in planta. Compared with the engineered *Klebsiella* strains without ACC deaminase activity, the engineered *Enterobacter* strains with ACC deaminase activity could reduce the plant ethylene levels. The engineered *Enterobacter* strains without IAA production showed the potentials to promote plant growth and wilt resistance.

## Conclusions

In this study, the variation of endosphere bacteriome and mycobiome of the banana plant during plant growth and wilting development suggested that the banana plant contained core bacterial and fungal taxa despite of plant physiological variation. The outgrowth of wilt pathogen did not occur during wilt development. The wilting symptom development was related with ethylene levels induced by pathogenic fungi. Although the keystone species *Enterobacter* sp. E5 did not show any activity against pathogenic fungi FOC4 in vitro, engineered *Enterobacter* sp. E5 with ACC deaminase activity on cells could promote banana plant growth and increase resistance to banana *Fusarium* wilt. Our results suggested that regulation of ethylene levels of banana plantlets by engineering plant core bacterial function may be of great interests to breeding banana resistant cultivars to *Fusarium* wilt. The novel strategies of engineering the interactions between endosphere microbiome with host plants provided valuable methods to build next-generation suitable agriculture.

## Additional files

Additional file 1: Figure S1, S2, and Table S1 Supplementary Table and Figures. (DOC 226 kb)
