# Supplementary material for: Engineering banana endosphere microbiome to improve Fusarium wilt resistance in banana
Source: Microbiome. 2019 May 15;7:74. doi: 10.1186/s40168-019-0690-x (PMC6521393; doi:10.1186/s40168-019-0690-x)
Supplement: Supplementary file 1 — Figure S1, S2, and Table S1 Supplementary Table and Figures. (DOC 226 kb) [file 40168_2019_690_MOESM1_ESM.doc]

**Engineering banana endosphere microbiome to improve Fusarium wilt resistance in banana**

Authors:

Yupei Liu^1^, Aiping Zhu^2^, Hongming Tan^2^, Lixiang Cao^2^*, and Renduo Zhang^1^*

^1^ School of Environmental Science and Engineering, Guangdong Provincial Key Laboratory of Environmental Pollution Control and Remediation Technology, Sun Yat-sen University, Guangzhou, China.

^2^ School of Life Sciences, Guangdong Provincial Key Laboratory for Climate Change and Natural Disaster Studies, Sun Yat-sen University, Guangzhou, China.

Correspondence

**Lixiang Cao**

E-mail: [lssclx@163.com](mailto:lssclx@163.com)

Correspondence

**Renduo Zhang**

E-mail: [zhangrd@mail.sysu.edu.cn](mailto:zhangrd@mail.sysu.edu.cn)

**Supplementary Table**

**Table S1.** The characteristics of effective tags from samples of pseudostems and roots of explants (designated as Sstem and Sroot), shoot tips and roots of healthy banana plants in fields without wilting symptoms (designated as Btip and Broot), shoot tips and roots of wilting banana plants (designated as Wtip and Wroot), and shoot tips and roots of banana plants adjacent to the wilting plants but without wilting symptoms (designated as Htip and Hroot).

| Sample | V3-V4 tags* | | | | ITS2 tags* | | | |
| --- | --- | --- | --- | --- | --- | --- | --- | --- |
|  | Numbers | Total length (bp) | Max length (bp) | Min length(bp) | Numbers | Total length (bp) | Max length (bp) | Min length (bp) |
| Sstem | 307 | 140872 | 482 | 401 | 9652 | 3147817 | 485 | 156 |
| Btip | 134 | 60911 | 487 | 408 | 16382 | 4555901 | 486 | 161 |
| Htip | 121 | 55445 | 467 | 414 | 12420 | 4060827 | 400 | 170 |
| Wtip | 83 | 37094 | 481 | 401 | 10156 | 3289254 | 485 | 153 |
| Sroot | 1020 | 468300 | 487 | 414 | 10911 | 3436804 | 486 | 151 |
| Broot | 1538 | 685502 | 467 | 401 | 22155 | 8080770 | 450 | 151 |
| Hroot | 11710 | 5271968 | 489 | 420 | 11990 | 4574712 | 450 | 151 |
| Wroot | 5099 | 2282729 | 487 | 420 | 20114 | 7323112 | 450 | 151 |

*V3-V4: V3-V4 hyper variable regions of bacterial 16S *rRNA* genes. ITS2: internal transcribed spacer 2 regions of fungal *rRNA* genes.

**Supplementary** **Figures**

**Fig. S1** The profiles of constructed plasmid pPIA.

**Fig. S2** (**A)** Effects of inoculation on growth of banana with treatments of the control (CK), *Kosakonia* sp. S1 (S1), *Enterobacter* sp. E5 (E5), *Klebsiella* sp. Kb (Kb), and the engineered strains of E5, S1, and Kb (designated as E5P, S1P, and KbP, resepectively). After the plantlets were grown in the FOC4 infested soils in pots for 90 d. (**B**) Effects of inoculation on *Fusarium wilt* resistance of banana with the different treatments. After the plantlets were grown in the FOC4 infested soils in pots for 90 d.

**Fig. S1**


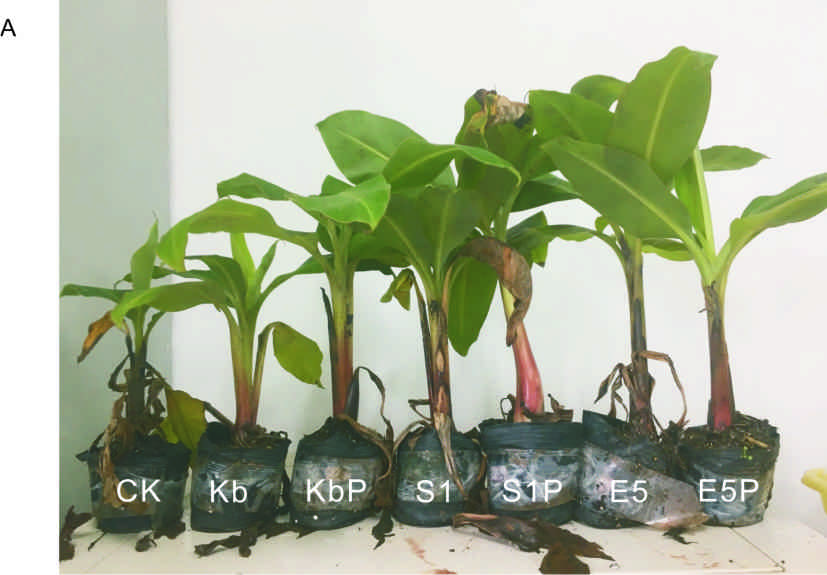


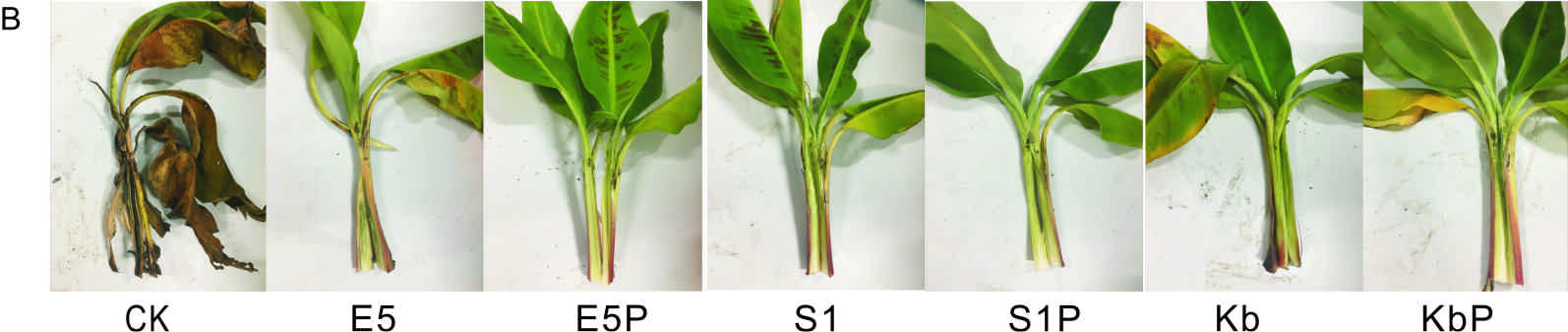


**Fig. S2**
